# An Experimental Study on 3D-Printed Gyroid-Shaped TC4 Porous Scaffolds Guiding Angiogenesis and Osteogenesis in Bone Defect Areas

**DOI:** 10.1021/acsbiomaterials.5c01845

**Published:** 2026-01-09

**Authors:** Lei Wang, Yu Wang, Rui Liu, Yanfeng Liang, Yang Liu, Mingqi Xu, Jia Yu, Yucheng Su, Zekui Han, Xinyu Wang

**Affiliations:** † Key Laboratory of Oral Biomedical Materials and Clinical Application, School of Stomatology, 117916 Jiamusi University, 522 Hongqi Street, Jiamusi 154002, China; ‡ Experimental Center of Stomatology Engineering, School of Stomatology, Jiamusi University, 522 Hongqi Street, Jiamusi 154002, China; § Life Science Key Laboratory Center of Basic Medicine, School of Basic Medicine, Jiamusi University, 258 Xuefu Street, Jiamusi 154007, China; ∥ Beijing Implant Training College (BITC), 109 Xidan North Street, Beijing 100032, China

**Keywords:** Gyroid-shaped, porous scaffold, 3D printing, angiogenesis, osteogenesis, tissue engineering

## Abstract

To investigate the ability of novel Gyroid-shaped titanium alloy (TC4) porous bioscaffolds to induce angiogenesis and osteogenesis in bone defect areas. This study employed selective laser melting (SLM) technology to fabricate Gyroid shaped and Cube-shaped TC4 porous bioscaffolds, using the commonly used cube shape as a control. The unit cell size was 4 mm, with a wall thickness or rod diameter of 300 μm and a porosity of approximately 80%. These scaffolds were implanted into rabbit mandibular defect sites (10 mm × 7 mm × 5 mm) to evaluate the angiogenic and osteogenic potential of the Gyroid-shaped scaffold. Material characterization revealed that sandblasted and acid-etched (SLA) TC4 scaffolds met design specifications, exhibiting uniformly distributed micrometer-scale pores and enhanced surface hydrophilicity. Histological staining revealed that compared to the Cube-shaped scaffold, the Gyroid-shaped scaffold induced greater angiogenesis and new bone formation within the bone defect area. Both scaffolds demonstrated good biocompatibility. Western Blot and RT-qPCR results indicated that the Gyroid-shaped scaffold possessed superior angiogenesis potential (compared to the Cube-shaped scaffold). During the early implantation phase (1–2 weeks), Gyroid-shaped scaffolds exhibited higher expression of platelet-endothelial cell surface adhesion molecule 1 (CD31) and endothelial mucin (EMCN). Concurrently, vessel distribution within the scaffold showed spatial variation. Additionally, gene expression of hypoxia-inducible factor 1α (HIF-1α) and vascular endothelial growth factor A (VEGFA) was elevated in the early bone defect area. Imaging analysis confirmed successful implantation of both scaffolds, with the Gyroid-shaped scaffold exhibiting a higher proportion of new bone formation. Consequently, the novel Gyroid-shaped TC4 porous bioscaffold demonstrates excellent potential for angiogenesis and osteogenesis, providing a reference for Gyroid-shaped scaffold-based bone defect repair.

## Graphical abstract

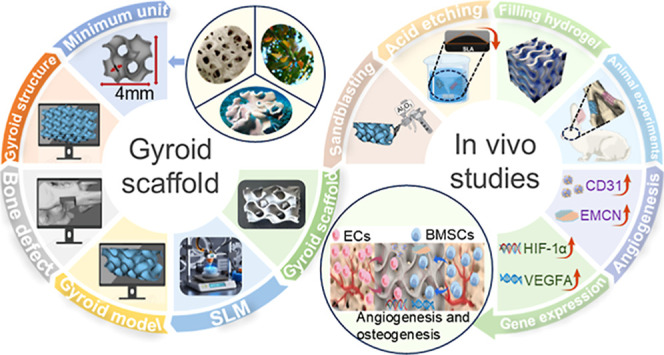

## 1 Introduction

The jawbone serves as the foundational structure supporting facial morphology and enabling masticatory function. Minor jawbone defects can be healed through the body’s self-repair mechanisms.^1^ However, extensive bone defects caused by severe infections, trauma, or tumors exceed the body’s self-repair capacity, leading to significant maxillofacial deformities and partial functional loss.^2,3^ This severely impacts patients’ quality of life and psychological well-being.^4^ Therefore, repairing extensive jawbone defects requires both restoring the original facial contour and reestablishing masticatory function, making such reconstructive efforts critically important.^5^ Furthermore, within the mammalian skeletal system, angiogenesis is intrinsically linked to bone remodeling processes. This is because blood vessels provide essential blood supply, nutrient delivery, and regulatory control for bone regeneration.^6,7^ Research indicates that regulating the formation of H-type vessels and their associated signaling pathways can effectively promote bone healing while reducing bone loss and the incidence of osteoarthritis.^8^

Currently, clinical methods for reconstructing extensive maxillofacial bone defects are primarily categorized based on bone source: autogenous bone, allogeneic bone, xenogeneic bone, and synthetic materials. Autologous bone, with its strong osteoinductive properties and low immunogenicity, is considered the “gold standard” for maxillofacial bone defect repair, exemplified by techniques such as vascularized fibular muscle-skin flaps and iliac crest bone grafts.^9,10^ However, autologous bone grafting still presents limitations, including restricted donor availability, compromise to donor site structure and function, suboptimal morphology, and prolonged surgical duration. Allogeneic and xenogeneic bone materials exhibit higher immunogenicity, potentially triggering immune rejection, infection, and disease transmission risks. Given these limitations, numerous researchers have explored more effective bone replacement materials in recent years. This led to the development of composite bone tissue engineering scaffolds, which consist of a biocompatible scaffold, stem cells, and exogenous growth factors, playing a crucial role in promoting bone tissue regeneration and repair.^11,12^ However, issues such as stem cell sourcing, maintaining cell viability, immune rejection, and high costs have limited their widespread application.^13,14^ Consequently, safe and reliable acellular tissue engineering composite scaffolds hold promise as novel alternative materials for bone regeneration. Nevertheless, current experimental research on acellular scaffolds without exogenous growth factors in promoting bone angiogenesis and regeneration remains relatively scarce.

With the continuous advancement of tissue engineering, various synthetic scaffold materials have been developed, including metals, bioceramics, and polymeric materials. Among these, titanium alloy (TC4) stands out as a widely used artificial bone substitute material. Possessing excellent biocompatibility, corrosion resistance, and mechanical properties, it is an ideal candidate for bone scaffolds.^15–17^ However, the biological inertness of the TC4 surface has, to some extent, limited its early bone integration capability.^18,19^ To resolve this issue, our previous work explored sandblasting and acid etching conditions for TC4 surfaces, identifying a treatment regimen that enhances its bone integration capacity.^20^ In fields spanning materials science, mechanics, and tissue engineering, triple-periodic minimal surfaces (TPMS) represent novel porous structures designed for lightweight and multifunctional applications, emerging as a research hotspot. Representative TPMS include Gyroid, Diamond, and Primitives.^21^ These structures feature high specific surface area, infinitely extendable smooth surfaces, and excellent energy absorption properties.^22,23^ Moreover, porous structures provide ideal growth spaces for tissue regeneration. Through finite element analysis and mechanical testing, we found that the elastic modulus (1.92 ± 0.29 GPa) and compressive strength (110.84 ± 1.14 MPa) of the Gyroid-shaped TC4 scaffold (unit cell size is 4 mm, wall thickness is 300 μm) match those of human mandible, effectively reducing stress shielding.^24^ Coculture with MC3T3-E1 cells demonstrated superior performance in cell adhesion, proliferation, and osteogenic differentiation compared to pillar structures.^25^ However, limited research exists on the angiogenic potential of Gyroid-shaped TC4 implants within bone defect sites, forming the focus of this study.

Traditional manufacturing processes have struggled to achieve high-precision fabrication of complex porous structures. The deepening application and increasing maturity of selective laser melting (SLM) technology in life sciences, coupled with the rising demand for personalized medical solutions, have opened new possibilities for designing and manufacturing complex porous structures through 3D printing.^26–28^ Previously, smaller unit cell dimensions were preferred to prevent fracturing of metallic lattice structures. However, in practical applications, both continuous surface structures and pillar structures require dimensions approaching the manufacturing resolution of the additive manufacturing platform to ensure fabrication accuracy.^29^ Therefore, this study employed SLM technology to fabricate a 4 mm unit cell-sized Gyroid-shaped TC4 porous scaffold. The scaffold surface was treated using SLA technology, followed by filling the pores with Poloxamer 407 hydrogel to construct a novel Gyroid-shaped TC4 porous bioscaffold. Material characterization and in vivo experiments evaluated the scaffold’s effects on angiogenesis and osteogenesis in bone defect areas (Figure 1).

**Figure 1:**
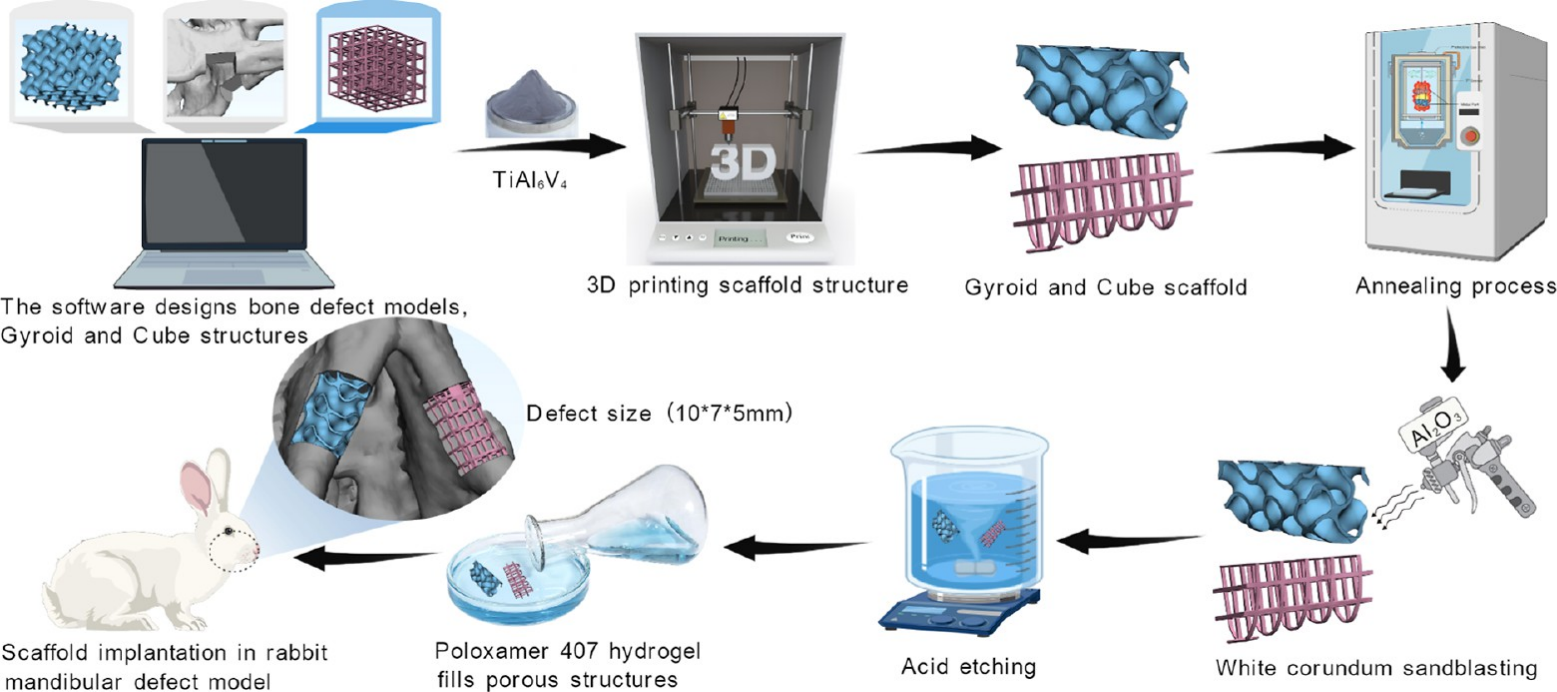
Schematic diagram of the design and fabrication of Gyroid-shaped and Cube-shaped TC4 porous bioscaffolds.

## 2 Materials and Methods

### 2.1 Scaffold Preparation

Using Mimics Research 21.0 (Materialise, Belgium) and Geomagic Wrap 2017 (3D System, USA) reverse engineering software, the “.DICOM” format data from the rabbit head and neck CT scan was processed to extract and refine the three-dimensional mandible model. The 3-matic Research 15.0 software (Materialise, Belgium) was applied to design bilateral mandibular defect models, each measuring 10.0 mm × 7.0 mm × 5.0 mm. The unit cell size for both the Gyroid and Cube structures was set to 4 mm, with a wall thickness or rod diameter of 300 μm. Since a single unit cell within the Gyroid structure contains multiple interconnected pores, a cross-shaped structure was inserted into the Cube structure to create a unit cell feature similar to that of the Gyroid structure. Based on the defect morphology and size, personalized scaffolds were constructed using boolean operations: a Gyroid-shaped (right) and a Cube-shaped (left). After adding supports using AutoFab MLab64 2.0 software (Renishaw, UK), the designs were imported into a metal 3D printer (Concept Laser, Germany) to fabricate the “Ti-6Al-4 V” scaffolds. Printing parameters: laser power 95W, scan speed 900 mm/s, layer thickness 25 μm, argon gas concentration ≥99.9%. Postprocessing involved annealing, followed by sandblasting acid etching (SLA) and ultrasonic cleaning (UC). Sandblasting conditions: 80-mesh white fused alumina (Al_2_O_3_), pressure 0.3 GPa, distance 4 cm, duration 10–20 s. Acid etching conditions: mixed acid solution (hydrochloric acid, sulfuric acid, and distilled water at a volume ratio of 1:1:2), temperature controlled at 75 ± 1 °C, etching time 30 min. The ultrasonic cleaning (UC) sequence was distilled water, acetone, anhydrous ethanol, and distilled water, each for 15 min. Finally, liquid poloxamer 407 hydrogel was filled into the pores of the Gyroid-shaped and Cube-shaped TC4 porous scaffolds. After heating, it converted to a gel state, completing the preparation of the Gyroid-shaped and Cube-shaped TC4 porous bioscaffolds.

### 2.2 Material Characterization

#### 2.2.1 Size Accuracy of the Scaffolds

Using a stereomicroscope (ZEISS, Germany), the wall thickness of the Gyroid-shaped TC4 scaffold and the rod diameter width of the Cube-shaped TC4 scaffold were measured, respectively. These measurements were compared with the design values (300 μm) to verify whether the machining dimensions of both scaffold structures met the design requirements.

#### 2.2.2 Pore Characteristics of the Scaffolds

We employed a nondestructive method to calculate the porosity of Gyroid-shaped and Cube-shaped TC4 porous scaffolds. The total volume of defects (*V*) was obtained using 3-matic software (262.01 mm^3^ on the left, 263.50 mm^3^ on the right). The actual mass (*m*) of the Gyroid-shaped and Cube-shaped TC4 porous scaffolds was weighed using an electronic analytical balance (Sartories, Germany), with density of Ti-6Al-4 V being 4.43 g/cm^3^(ρ). The actual porosity of both scaffold structures was calculated using Formula [Disp-formula eq1] and Formula [Disp-formula eq2] for five replicate samples each.

1 $$Actual\;porosity=\left(1-V_{1}/V\right)\times 100\;\%$$

2 $$V_{1}=m/\rho$$

Additionally, we measured the actual pore size of the Gyroid-shaped and Cube-shaped TC4 porous scaffolds using an optical microscope and obtained the specific surface area of both scaffold structures via design software.

#### 2.2.3 Surface Characteristics of the Scaffolds

Surface morphology observation and elemental analysis of Gyroid-shaped and Cube-shaped TC4 porous scaffolds were conducted using field emission scanning electron microscopy (SEM, ZEISS, Germany) and energy dispersive X-ray spectroscopy (EDS, ZEISS, Germany). Samples were randomly grouped into the ultrasonic cleaning (UC), sandblasting (SA), and sandblasting-acid etching (SLA) groups. After securing the scaffolds to the sample stage with conductive adhesive, they were placed in the vacuum chamber. Parameters such as mode and magnification were adjusted to observe and photograph the microscopic morphology of the scaffold surfaces. Concurrently, characteristic X-ray signals were collected to analyze the content and distribution of elements including titanium (Ti), aluminum (Al), vanadium (V), oxygen (O), carbon (C), and silicon (Si), generating corresponding spatial distribution maps for each element.

#### 2.2.4 Contact Angle Measurement

Place 2 μL of distilled water onto the titanium sheet. The water contact angle of the titanium sheets in the UC, SA, and SLA groups was measured using a contact angle measuring instrument (Power Each Company, China), and the images were recorded.

#### 2.2.5 Degradation of Poloxamer 407 Hydrogel

The initial mass (*W*_0_) of the gelatinous poloxamer 407 hydrogel was weighed. It was then placed in phosphate-buffered saline (PBS, pH = 7.4) at 37 °C. The gel was removed and weighed (*W*_*x*_) every 0.5 or 1 h. The percentage of remaining poloxamer 407 hydrogel was calculated using eq 3.

3 $$Remaining\;percentage=W_{X}/W_{0}\times 100\;\%$$

### 2.3 In Vivo Animal Studies

#### 2.3.1 Surgical Procedure

This study was reviewed and approved by the Animal Ethics Committee of the Affiliated Stomatological Hospital of Jiamusi University (Ethics No.: KQYXY-2025-XS-H029). Fifty-four healthy male Chinese white rabbits (weight 2.5–3.5 kg, age 4 months) were randomly assigned to groups based on different observation periods (1, 2, 3, 4, 6, 8, 12 weeks) for bilateral mandibular defect model construction and scaffold implantation experiments. Rabbits were anesthetized via ear vein injection of 2% sodium pentobarbital (30–60 mg/kg). They were fixed in the supine position, with the surgical site routinely disinfected and draped, followed by local injection of lidocaine solution. A skin incision was made along the mandibular body to expose the bone surface. A reciprocating saw was used to create bone defects of the designed dimensions (10 mm × 7 mm × 5 mm), with continuous saline irrigation for cooling during the procedure. Subsequently, Gyroid-shaped and Cube-shaped TC4 porous bioscaffolds were implanted into the right and left mandibular defects, respectively, and secured in place. After thorough hemostasis, the incisions were sutured (Figure 2A,B). Postoperatively, the surgical site was disinfected with povidone-iodine for 7 consecutive days, and cefazolin sodium (50–100 mg/kg) was administered intramuscularly for infection prevention.

**Figure 2:**
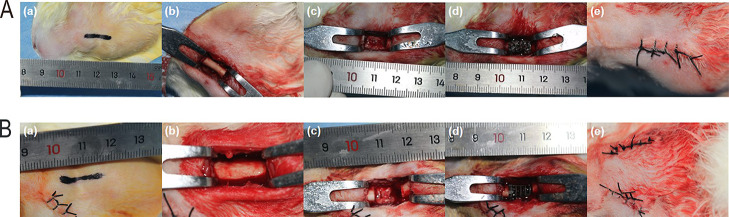
(A,B) Surgical procedure for implantation of (A) Gyroid-shaped and (B) Cube-shaped TC4 porous bioscaffolds.

#### 2.3.2 Biocompatibility Testing

Rabbits without scaffold implantation and those implanted with scaffolds at 4, 8, and 12 weeks were euthanized, and heart, liver, spleen, lung, and kidney tissues were collected. Samples were fixed in 10% formalin solution for 48 h, followed by dehydration, clearing, paraffin immersion, and embedding. Four-micrometer slices were prepared for hematoxylin and eosin (H&E) staining. The biological safety of the scaffolds was assessed through observation under an optical microscope (Olympus, Japan).

#### 2.3.3 Histological Analysis

At 4, 8, and 12 weeks postimplantation, bilateral mandibular samples were extracted. After 48 h of fixation, hard tissue sections underwent hematoxylin and eosin (H&E) staining following the previously described procedure, with section thickness approximately 30 μm. New blood vessel formation and new bone tissue development within regions of interest (ROI) were observed using an optical microscope, and quantitative analysis was performed using ImageJ software.

#### 2.3.4 Western Blotting

Experimental rabbits were euthanized at different observation time points. Bilateral mandibular scaffold specimens were extracted using sterile surgical instruments (high-pressure steam sterilization). A hard tissue microtome (with blades disinfected in alcohol for 1 day, EXAKT, Germany) was employed to divide the scaffold structure into four equal segments (upper front, lower front, upper back, lower back in anatomical position). Simultaneously, the specimens were cooled by rinsing with 4 °C DEPC-treated water. Hemostatic forceps or needle holders were then used to apply external force to the scaffold structure, causing deformation or fracture of the metal porous scaffold. Newly formed tissue within the scaffold was gently scraped out using a metal probe and immediately placed into 2 mL cryovials for storage in liquid nitrogen. The entire process is conducted on ice to prevent protein denaturation.

Total protein was extracted from the liquid nitrogen-ground tissue using RIPA lysis buffer (Beyotime, China) with PMSF protease inhibitor (Beyotime, China). Protein concentration was measured using a microplate reader, and sample loading buffer was prepared. Samples containing equal total protein amounts underwent SDS-PAGE (Boster, USA) electrophoresis at 80 V for 30 min and 120 V for 1 h. Subsequently, proteins were transferred to PVDF membranes (Millipore, USA) and blocked with 5% nonfat milk for 1 h. Primary antibodies CD31 (Proteintech, 1:5000), ECMN (Proteintech, 1:3000), and β-actin (Zhongshan Golden Bridge, 1:2000) were added separately and incubated overnight at 4 °C (≥12 h). The membrane was washed in TBST buffer, then incubated at room temperature with secondary antibody (Boster, 1:15,000) for 1 h. Finally, target proteins were detected using ECL chemiluminescence (Meilunbio, China). The β-actin protein served as an internal control.

#### 2.3.5 Real-Time Quantitative PCR

Following the previously described method, newly formed bone tissue within the two types of scaffolds was extracted at different observation time points. After grinding with liquid nitrogen, total RNA was extracted using commercial TRIzol reagent (Invitrogen, USA). RNA was reverse transcribed into cDNA using the PrimeScript RT Reagent Kit (Takara, Japan). Real-time quantitative PCR was performed using the TB Green (Takara, Japan) chimeric fluorescent method and the Applied Biosystems 7300 PCR Detection System (Applied Biosystems, USA). Relative expression levels of hypoxia-inducible factor 1α (HIF-1α) and Vascular Endothelial Growth Factor A (VEGFA) genes were analyzed using the 2^^−ΔΔCT^ method, with GAPDH as the internal reference gene. Primer sequences used in the experiment are listed in Table 1.

**Table 1:** Primers Used for Real-Time Quantitative PCR

target gene	forward primer (5′–3′)	reverse primer (5′–3′)
HIF-1α	GCATCTCCGTCTCCTAACCA	ACACGTTAGGGCTTCTTGGA
VEGFA	GCTGCTGCAATGATGAAAGC	CTGCATGGTGACGTTGAACT
GAPDH	GTCGGAGTGAACGGATTTGG	TTGATGGCGACAACATCCAC

#### 2.3.6 CBCT Assessment

Cone-beam computed tomography (CBCT, Sirona, Germany) scans were performed on experimental rabbits at 4, 8, and 12 weeks postimplantation for both types of scaffolds. Image data in “.DICOM” format were processed using Mimics Research 21.0 and Geomagic Wrap 2017 software to extract new bone formation within the scaffolds. The volume of new bone was then obtained using 3-matic Research 15.0 software. The new bone volume fraction (NBV/TV) was determined based on the total defect volume to evaluate the bone regeneration capacity and implant efficacy of the Gyroid-shaped TC4 porous bioscaffold.

### 2.4 Statistical Analysis

Data for each group were expressed as mean ± standard deviation (SD). Differences between groups were analyzed using *t* tests or one-way analysis of variance (ANOVA). Statistical analysis and graphing were performed using SPSS 27.0 (IBM, USA) and GraphPad Prism 9.1 (GraphPad, USA). *P* < 0.05 indicates statistically significant differences, while *P* > 0.05 indicates no statistically significant differences.

## 3 Results

### 3.1 Scaffold Characterization

#### 3.1.1 Structural Features of the Scaffolds

Stereomicroscopic measurements revealed that the average wall thickness of the Gyroid-shaped TC4 porous scaffold was 301.12 ± 2.35 μm (Figure 3Ba), while the average rod diameter of the Cube-shaped TC4 porous scaffold was 299.21 ± 0.87 μm (Figure 3Bb). Both values were close to the design dimensions (300 μm, Table 2), with no statistically significant difference (*P* > 0.05) (Figure 3C). Table 2 also shows that the porosities of the Gyroid-shaped and Cube-shaped TC4 porous scaffolds were 81.15 ± 0.33% and 86.24 ± 0.42%, with pore sizes of 996.87 ± 9.86 μm and 1357.16 ± 8.64 μm, and specific surface areas of 431.88 mm^2^ and 298.48 mm^2^, respectively.

**Figure 3:**
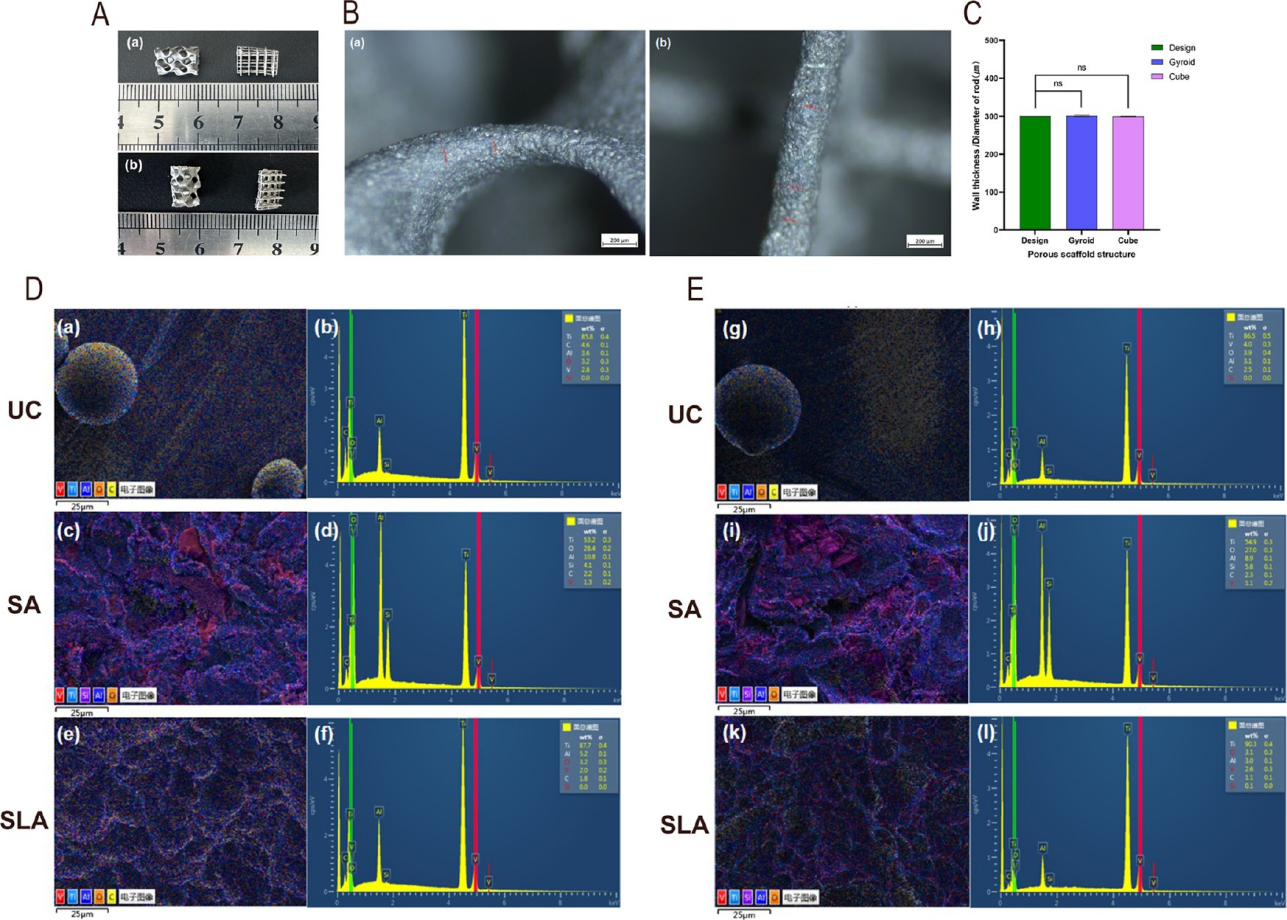
(A) Prepared Gyroid-shaped and Cube-shaped TC4 porous scaffolds. (B) Representative images for dimensional measurement of the (a) Gyroid-shaped scaffold and (b) Cube-shaped scaffold. (C) Dimensional accuracy analysis of Gyroid-shaped and Cube-shaped scaffold, *n* = 5 per group. *P < 0.05, **P < 0.01, ***P < 0.001, ****P < 0.0001, ns indicates no statistically significant difference. (D,E) Surface element content and spatial distribution maps for the (D) Gyroid-shaped scaffold and (E) Cube-shaped scaffold after UC, SA, and SLA treatments.

**Table 2:**
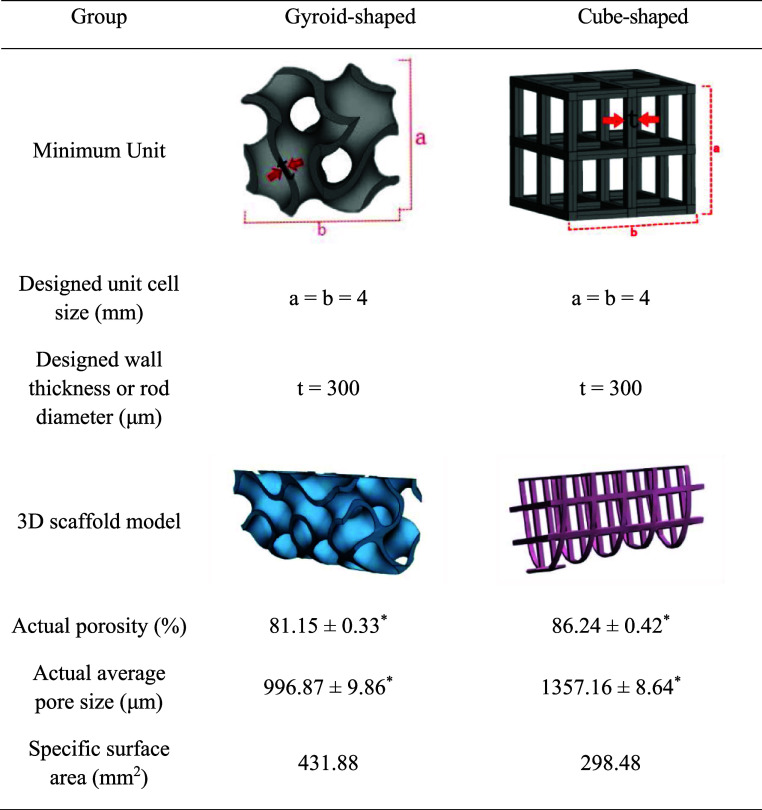
Structural Parameters and 3D Models of Gyroid-Shaped and Cube-Shaped TC4 Porous Scaffolds

Note: *n = 5 per group.

#### 3.1.2 Elemental Composition of the Scaffolds

EDS surface scanning results indicated that after sandblasting (SA) treatment, the surface Al and O elemental content of both scaffolds significantly increased, while the relative proportions of other elements decreased (Figure 3D­(c,d),E­(i,j)). Following acid etching, the Al and O content decreased, and the elemental ratios recovered to levels close to the initial state, similar to the results of the ultrasonic cleaning (UC) group (Figure 3D­(a,b,e,f),E­(g,h,k,l)).

#### 3.1.3 Surface Topography of the Scaffolds

SEM observations showed that the surfaces of Gyroid-shaped and Cube-shaped TC4 porous scaffolds printed using selective laser melting (SLM) technology retained a large number of unfused titanium alloy (TC4) particles and exhibited defective step structures (Figure 4A­(a–c),B­(j–l)). Following sandblasting (SA) treatment, residual particles and step structures were largely eliminated, revealing a locally smooth, lamellar morphology (Figure 4A­(d–f),B­(m–o)). Subsequent acid etching resulted in a uniform micrometer-scale porous surface morphology on the scaffold (Figure 4A­(g–i),B­(p–r)).

**Figure 4:**
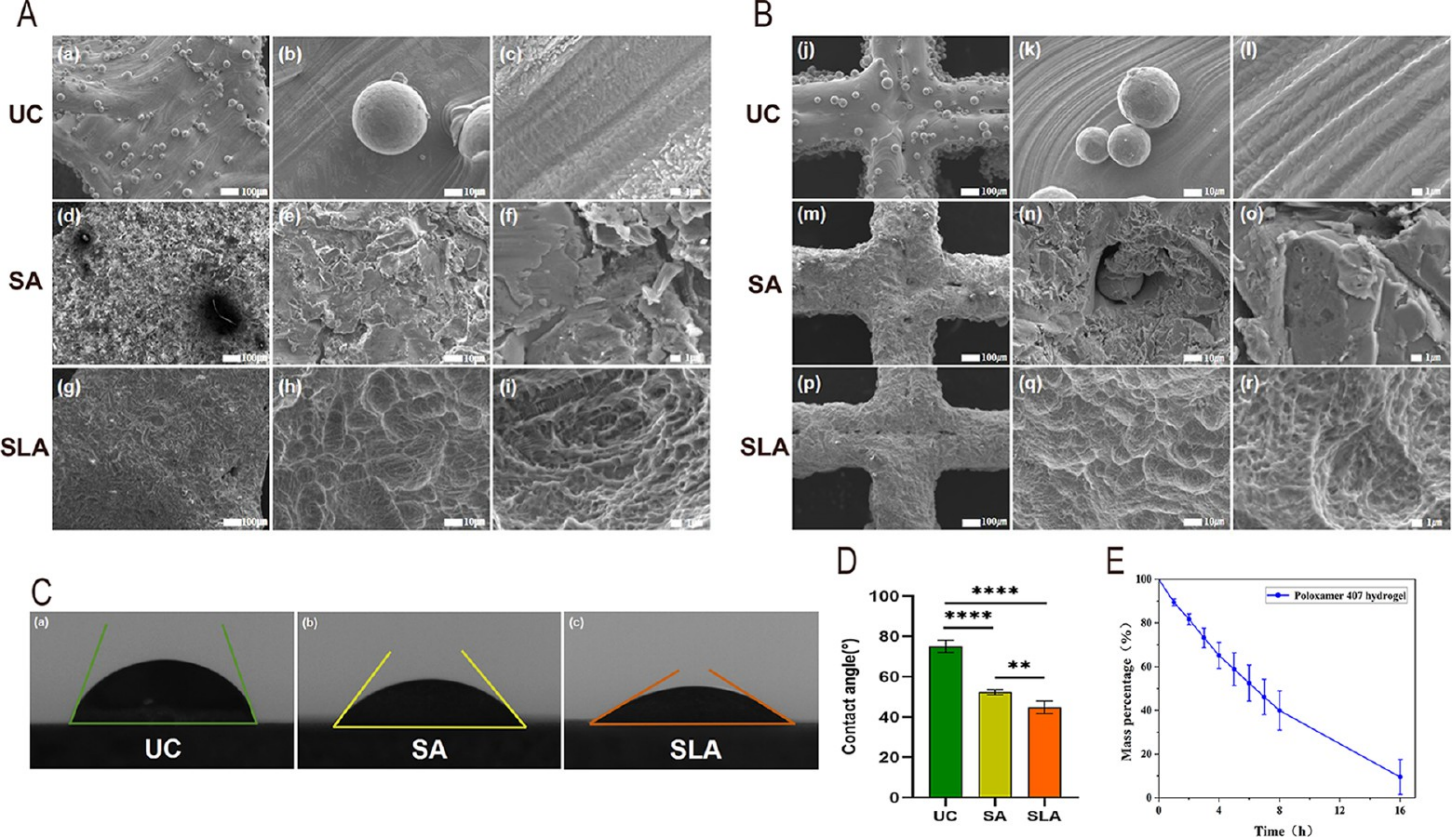
(A,B) SEM observation of surface morphology images for (A) Gyroid-shaped and (B) Cube-shaped TC4 scaffolds. (A­(a–c),B­(j–l)) After ultrasonic cleaning (UC); (A­(d–f),B­(m–o)) After sandblasting (SA); (A­(g–i),B­(p–r)) After sandblasting-acid etching (SLA). (A­(a,d,g),B­(j,m,p)) at 100× magnification, (A­(b,e,h),B­(k,n,q)) at 1000× magnification, (A­(c,f,i),B­(l,o,r)) at 6000× magnification. (C) Water contact angles on TC4 surfaces treated with (a) UC, (b) SA, and (c) SLA. (D) Statistical analysis of water contact angles, *n* = 5 per group. (E) Degradation profile of poloxamer 407 hydrogel, *n* = 4 per group. *P < 0.05, **P < 0.01, ***P < 0.001, ****P < 0.0001, ns indicates no statistically significant difference.

#### 3.1.4 Contact Angle

The average water contact angles for the UC, SA, and SLA groups were 75.03° ± 3.02°, 52.34° ± 1.28°, and 44.80° ± 3.12°, respectively (Figure 4C). Comparisons among groups revealed statistically significant differences (*P* < 0.05) (Figure 4D), indicating that SLA treatment significantly enhances the hydrophilicity of the TC4 scaffold surface.

#### 3.1.5 Degradation of Poloxamer 407 Hydrogel

In vitro degradation experiments indicated that poloxamer 407 hydrogel underwent gradual degradation over time in phosphate-buffered saline (PBS, pH 7.4) at 37 °C. Approximately 20% degradation occurred within 2 h, reaching about 50% degradation at 6 h, with cumulative degradation reaching approximately 90% by 16 h (Figure 4E).

### 3.2 In Vivo Animal Studies

#### 3.2.1 Biocompatibility Analysis

Compared with the group without scaffold implantation, rabbits implanted with Gyroid-shaped and Cube-shaped TC4 porous bioscaffolds for 4, 8, and 12 weeks showed no significant pathological changes such as inflammatory reactions, abnormal proliferation, or necrosis in H&E-stained heart, liver, spleen, lung, and kidney tissues (Figure 5A). This indicated that both types of TC4 porous bioscaffolds exhibited excellent biocompatibility.

**Figure 5:**
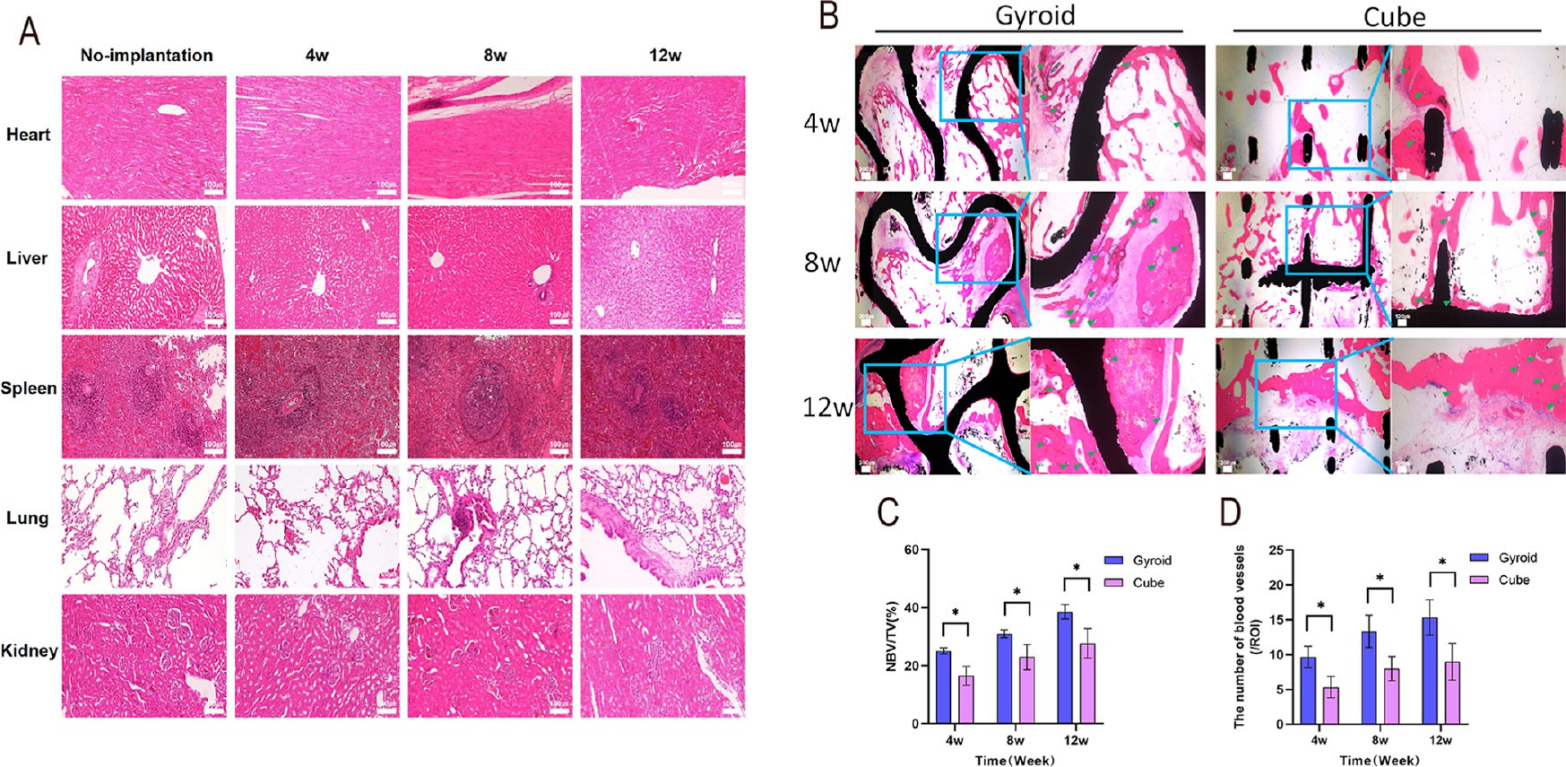
(A) H&E staining of rabbit visceral tissues implanted with Gyroid-shaped and Cube-shaped scaffold at 4, 8, and 12 weeks compared to non-implanted controls. (B) H&E staining of hard tissue sections from rabbits implanted with both scaffolds at 4, 8, and 12 weeks. The left side shows the overall image at each time point, and the right side shows the partial image. (The red area is new bone formation within the scaffold, the black area is the TC4 scaffold, the green arrows represent new blood vessels, and the white area is the gap in the non-ingrown tissue). (C) Quantitative analysis of NBV/TV ratio for different scaffolds at 4, 8, and 12 weeks. (D) Quantitative analysis of the number of blood vessels within the material in a single field of view. *n* = 3 per group. *P < 0.05, **P < 0.01, ***P < 0.001, ****P < 0.0001, ns indicates no statistically significant difference.

#### 3.2.2 Histological Analysis

Histological findings of vascular and bone formation following implantation of the two scaffolds were shown (Figure 5B). At 4 weeks post-implantation, newly formed bone tissue exhibited discontinuous distribution within both the Gyroid-shaped and Cube-shaped scaffolds, primarily localized at the scaffold–bone interface, with a limited number of new blood vessels. At 8 weeks, the initially scattered bone tissue within both scaffolds gradually connected to form partially continuous trabecular structures extending into the scaffold interior. Both the proportion of new blood vessels and bone tissue increased compared to 4 weeks. After 12 weeks of implantation, relatively complete and tightly connected bone tissue formation was observed surrounding both scaffolds, though the growth rate of new blood vessels leveled off. Quantitative analysis revealed that at 4, 8, and 12 weeks post-implantation of the Gyroid-shaped scaffold, the proportion of new bone area within the region of interest (ROI) was 25.12 ± 1.03%, 31.02 ± 1.35%, and 38.51 ± 2.46%, respectively. The number of new blood vessels was 9.67 ± 1.53, 13.33 ± 2.31, and 15.33 ± 2.52, respectively. In contrast, the Cube-shaped scaffold exhibited new bone area proportions of 16.55 ± 3.30%, 23.02 ± 4.33%, and 27.72 ± 5.07%, with the number of new blood vessels being 5.33 ± 1.53, 8.00 ± 1.73, and 9.00 ± 2.65, respectively. Statistical analysis indicated that the Gyroid-shaped TC4 porous bioscaffold significantly outperformed the Cube-shaped scaffold in promoting both new bone and new blood vessel formation (*P* < 0.05) (Figure 5C,D).

#### 3.2.3 Angiogenesis within the Scaffolds

Western blot results showed that the expression levels of platelet-endothelial cell surface adhesion molecule 1 (CD31) and endothelial mucin (EMCN) in Gyroid-shaped scaffolds at different time points were significantly higher than those in the Cube-shaped scaffold group (*P* < 0.05) (Figure 6A,C,D). Further analysis at different time points after implantation of the Gyroid-shaped scaffold revealed that CD31 and ECMN exhibited higher expression as early as 1 week post-implantation, with expression levels significantly higher than those at 2–4 weeks (*P* < 0.05), while no significant difference was observed compared to 8 weeks and 12 weeks (*P* > 0.05) (Figure 6B,E,F). Additionally, regional analysis of the Gyroid-shaped scaffold revealed that during early angiogenesis (1–4 weeks), CD31 and EMCN expression was significantly higher in the upper bone defect region than in the mandibular border region (*P* < 0.05) (Figure 6G–L), indicating spatial distribution differences in vascularization.

**Figure 6:**
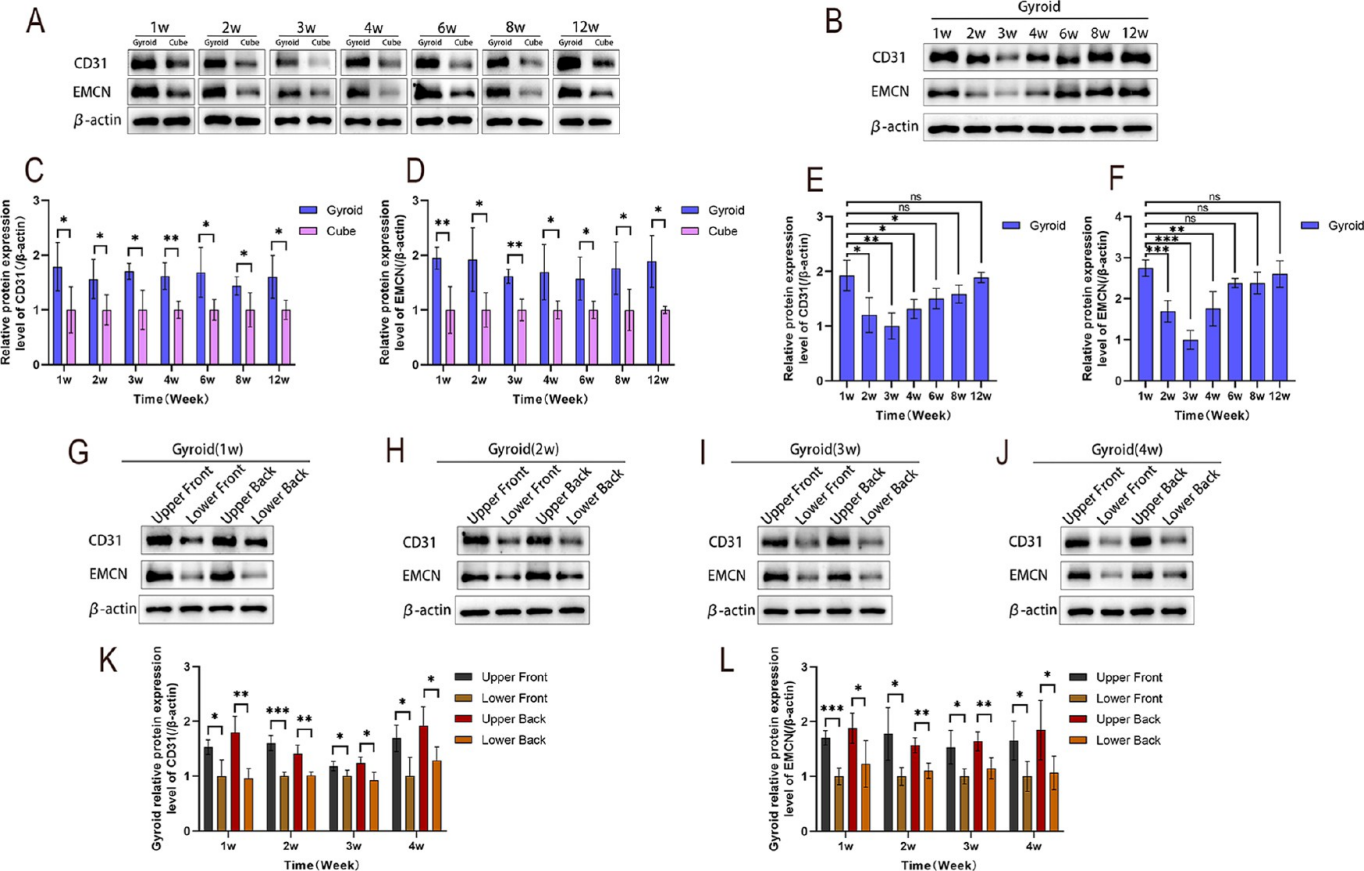
(A) Protein levels of CD31 and EMCN in Gyroid-shaped and Cube-shaped scaffolds at 1–12 weeks. (B) Protein levels of CD31 and EMCN in Gyroid-shaped scaffolds at different time points. (C) Quantitative analysis of CD31 levels in both scaffolds. (D) Quantitative analysis of EMCN levels in both scaffolds. (E) Quantitative analysis of CD31 levels in the Gyroid scaffold. (F) Quantitative analysis of EMCN levels in the Gyroid scaffold. (G–L) Distribution and quantitative analysis of (K) CD31 and (L) EMCN levels within the Gyroid-shaped scaffold at (G) 1 week, (H) 2 weeks, (I) 3 weeks, and (J) 4 weeks. *n* = 4 per group. *P < 0.05, **P < 0.01, ***P < 0.001, ****P < 0.0001, ns indicates no statistically significant difference.

#### 3.2.4 Expression of Angiogenesis-Related Genes

RT-qPCR results showed that at 1, 3, and 4 weeks postimplantation, the gene expression levels of hypoxia-inducible factor 1α (HIF-1α) and vascular endothelial growth factor A (VEGFA) in the Gyroid-shaped scaffold were significantly higher than those in the Cube-shaped scaffold group (*P* < 0.05). However, there was no significant difference between the two groups at 2 weeks (*P* > 0.05) (Figure 7A,B). Furthermore, results from different time points after Gyroid-shaped scaffold implantation showed that HIF-1α and VEGFA gene expression was higher at 1 week postimplantation, with expression levels significantly higher than at 3 and 4 weeks (*P* < 0.05), while no statistically significant difference was observed compared to 6–12 weeks (*P* > 0.05) (Figure 7C,D). This trend was consistent with Western Blot detection results.

**Figure 7:**
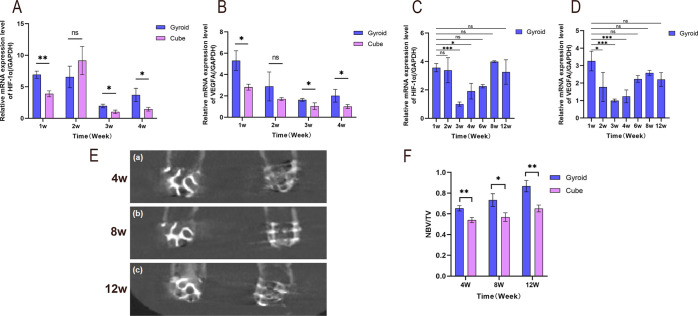
(A,B) Quantitative analysis of Gyroid-shaped and Cube-shaped scaffolds on (A) HIF-1α and (B) VEGFA gene expression at 1–4 weeks. (C,D) Quantitative analysis of Gyroid-shaped scaffolds on (C) HIF-1α and (D) VEGFA gene expression at different time points. (E) Representative CBCT images at 4, 8, and 12 weeks post-implantation for both scaffolds. (F) Quantitative analysis of NBV/TV from CBCT data. *n* = 3 per group. *P < 0.05, **P < 0.01, ***P < 0.001, ****P < 0.0001, ns indicates no statistically significant difference.

#### 3.2.5 CBCT Analysis

CBCT images revealed successful implantation of both scaffolds, with gradual increase in new bone formation. The Gyroid-shaped scaffold demonstrated significantly superior outcomes compared to the Cube-shaped scaffold (Figure 7E). Quantitative analysis at 4, 8, and 12 weeks showed new bone volume fraction (NBV/TV) values of 0.65 ± 0.03, 0.73 ± 0.06, and 0.87 ± 0.05 for the Gyroid-shaped scaffold; while those for the Cube-shaped scaffold were 0.54 ± 0.03, 0.57 ± 0.04, and 0.65 ± 0.03, respectively. This indicated significantly higher new bone volume within the Gyroid-shaped scaffold compared to the Cube-shaped scaffold group (*P* < 0.05) (Figure 7F), further confirming the superiority of the Gyroid-shaped scaffold in promoting bone regeneration.

## 4 Discussion

Autologous bone grafting possesses excellent osteoinductive properties and low immunogenicity, long regarded as the “gold standard” for clinical repair of maxillofacial bone defects. However, issues such as limited donor sources and secondary damage to donor sites severely restrict its further development.^30^ This has spurred rapid progress in bone tissue engineering, with titanium alloy (TC4) scaffold materials gaining significant attention due to their excellent biocompatibility, controllable mechanical properties, and superior osteogenic potential.^16^ Beyond the material itself, the scaffold’s topological structure plays a crucial role in regulating cell behavior and guiding angiogenesis and bone regeneration.^31^ Research indicated that composite scaffolds with a “flowerbed” structure, created by integrating 3D printing and electrospinning, could simulate the microenvironment of natural bone, effectively promoting angiogenesis and bone regeneration, offering new insights for scaffold design.^32^ Therefore, we propose that an ideal bone repair scaffold should possess porous features mimicking natural trabeculae, excellent mechanical properties, and the ability to regulate cellular biological behavior.^33,34^ Triple-periodic minimal surfaces (TPMS) represent a novel porous configuration with zero mean curvature. These structures not only share characteristics with natural bone trabeculae but also exhibit geometric similarities to coral, butterfly wings, and plant leaves found in nature. Among these, the Gyroid-shaped stands as a representative example. Bouakaz et al. discovered that Gyroid-shaped scaffolds made of hydroxyapatite (HA) possessed certain osteogenic potential, though their load-bearing capacity was relatively poor.^35^ The Gyroid-shaped had also been applied to the design of orthopedic fixation screws, demonstrating superior effects in osseointegration.^36^ Through finite element analysis and mechanical testing, we found that a 4 mm single-cell size, 300 μm wall thickness Gyroid-shaped TC4 porous scaffold exhibited elastic modulus (1.92 ± 0.29 GPa) and compressive strength (110.84 ± 1.14 MPa) comparable to human mandible.^24^ Coculture experiments with MC3T3-E1 cells demonstrated that the Gyroid-shaped scaffold promoted cell adhesion, proliferation, and osteogenic differentiation.^25^ Additionally, Li et al. reported that the Gyroid-shaped also enhanced the survival and proliferation of HUVECs.^31^ Therefore, this Gyroid-shaped scaffolding holds promise as a novel option for bone tissue engineering scaffold design. The cube-shaped was a commonly used configuration for pillar-type porous structures, which had been validated in multiple in vivo and in vitro studies to possess certain angiogenic and osteogenic capabilities.^37^ Therefore, it was designated as the control group in this study. Given that a single cell within a Gyroid structure contains multiple interconnected pores, we insert a cross-shaped structure into a cube structure (with a 4 mm single-cell size and 300 μm rod diameter) to create a single-cell feature similar to the Gyroid structure. This not only enhanced the mechanical properties of the Cube-shaped scaffold, but research also indicated that tissue deposition rates within cross-shaped pore scaffolds were twice as fast as those within square-shaped pore scaffolds.^38^

This study successfully fabricated Gyroid-shaped and Cube-shaped TC4 porous scaffolds with a 4 mm unit cell size and a wall thickness or rod diameter of 300 μm using selective laser melting (SLM) technology (Figure 3A). Both porous scaffolds met our design requirements. Beyond unit cell size and wall thickness, porosity and pore size are also critical parameters in porous scaffold design and application. Reviewing previous studies, porous TC4 implant designs primarily employed porosities ranging from 25% to 90% and pore sizes between 200 and 1500 μm.^39^ Our calculations revealed that the actual porosities of the Gyroid-shaped and Cube-shaped TC4 porous scaffolds used in this experiment were comparable, approximately 81%–86% (Table 2). However, some studies suggested that porosity exceeding 80% could compromise scaffold strength.^17^ This view is not absolute, as our prior mechanical testing confirmed that the Gyroid-shaped TC4 porous scaffold with a 4 mm unit cell size and 300 μm wall thickness met the strength requirements for the mandible.^24^ Moreover, the unique hyperbolic topology of the Gyroid-shaped endows it with significant energy-absorbing properties, effectively mitigating this concern.^23^ For the Cube-shaped scaffold, we incorporated a cross-shaped structure at its center, similarly ensuring its effective mechanical performance. Additionally, the pore sizes of the two porous scaffolds exhibit certain differences. The pore sizes of the Gyroid-shaped and Cube-shaped TC4 porous scaffolds were 996.87 ± 9.86 μm and 1357.16 ± 8.64 μm, respectively (Table 2), both exceeding the 600 μm pore size proposed by Taniguchi et al. for porous structures capable of inducing osteogenesis.^40^ Fukuda et al. also reported significant bone induction by porous TC4 implants with pore sizes of 500 and 600 μm.^41^ However, Wu et al. found that the geometric configuration of porous scaffolds influenced early angiogenesis in defect sites more significantly than pore size.^42^ Similarly, Arnal-Pastor et al. suggested that sponge-like porous scaffolds possessed greater angiogenesis potential than grid-like porous scaffolds.^43^ SEM analysis revealed substantial residual titanium alloy powder (TC4 microspheres) on the surface of TC4 porous scaffolds fabricated via selective laser melting. While surface treatments like ultrasonic cleaning or sandblasting acid etching can remove residual metal powder from large-pore scaffolds, this proves challenging for small-pore structures.^44^ Bai et al. observed that as pore size increases, the interconnectedness between pores expands, leading to greater numbers and larger diameters of internally grown vessels.^45^ Such large-pore scaffolds provide ample space for vessel ingrowth and osseointegration, facilitating mesenchymal stem cell adhesion, proliferation, and differentiation. This enables biological fixation between the implant and host tissue, enhancing long-term stability.^46,47^ Additionally, in bone tissue engineering scaffold design, similar porosities can create different specific surface areas.^48^ Compared to Cube-shaped scaffold, the Gyroid-shaped TC4 porous scaffold exhibits a larger specific surface area (Table 2), which is determined by the specific curvature characteristics of the Gyroid shape. Research indicated that moderately increasing implant surface area enhanced mechanical interlocking at the bone-scaffold interface, reduced micromovement of the scaffold, facilitated inward bone growth, and accelerated the osseointegration process.^49^ Surface modification techniques are also widely employed to enhance the biological properties of metallic implants. Sandblasting and acid etching (SLA) treatment retained the scaffold’s elemental composition and content while creating a uniform, rough micrometer-scale pore morphology on its surface. This effectively increased the scaffold’s microscopic surface area and hydrophilicity. The enhanced surface hydrophilicity facilitates the adhesion and proliferation of osteoblasts and vascular endothelial cells, thereby increasing the scaffold’s bioinductive activity. Zheng et al. concluded that SLA-treated 3D-printed TC4 implants exhibit improved surface morphology, wettability, and roughness, potentially enhancing their osteogenic promotion capacity without compromising the material’s overall mechanical properties.^20^

Based on the safety, thermosensitivity, and degradability of poloxamer 407 hydrogel, studies have utilized it as a carrier for stem cells or bioactive factors to simulate the extracellular matrix microenvironment, thereby effectively promoting angiogenesis and osteogenesis.^50^ We filled the pores of the scaffold with poloxamer 407 hydrogel without any additional components, serving as a temporary hemostatic agent to block surrounding tissue exudation and reduce infection risk. After 16 h of immersion in simulated body fluid, only approximately 10% of the hydrogel remained, providing ample space for vascular and bone tissue ingrowth. Following hydrogel filling, we successfully constructed Gyroid-shaped and Cube-shaped TC4 porous bioscaffolds. Although in vitro characterization confirmed the scaffolds’ favorable surface properties, their performance must be evaluated within the complex biological environment of the in vivo setting. Therefore, we established a rabbit mandibular defect model and implanted Gyroid-shaped and Cube-shaped TC4 porous bioscaffolds to further investigate the influence of Gyroid structures on angiogenesis and osteogenesis within in vivo bone defect sites.

Biological safety is a prerequisite for implant applications.^51^ Following implantation of both TC4 porous bioscaffolds, H&E staining of major organs revealed no significant toxic reactions. This is consistent with Hanawa et al.’s conclusion that TC4 exhibits excellent biocompatibility from the perspective of material surface properties, confirming the inherent safety of the material.^52^ Histological staining revealed significantly higher quantities of new bone and new blood vessels within the Gyroid-shaped TC4 porous bioscaffold compared to the Cube-shaped scaffold. This indicated that the Gyroid structure possessed superior angiogenesis potential and osteogenic capacity. It is well-known that osteogenesis and angiogenesis are spatially mutually reinforcing processes, a relationship termed the “angiogenesis-osteogenesis coupling”.^53^ Osteogenesis depends on effective angiogenesis, making prior vascular ingrowth crucial.^7,54^ New blood vessels supply essential nutrients, bioactive factors, and various functional cells for bone regeneration.^55^ Recent studies revealed that H-type vessels were closely associated with osteogenesis, exhibiting strong positive expression of platelet-derived endothelial cell surface adhesion molecule 1 (CD31) and endothelial mucin (EMCN).^8,56^ Our experiments showed significantly higher expression of CD31 and EMCN in Gyroid-shaped TC4 porous bioscaffolds compared to Cube-shaped scaffolds. Moreover, Gyroid-shaped scaffolds exhibited elevated CD31 and EMCN expression even during early implantation. This may be attributed to the Gyroid structure’s specific morphology, which possesses a higher specific surface area.^48^ This facilitates extensive adhesion of vascular endothelial cells to the scaffold surface during early implantation, creating favorable conditions for early vascular formation and subsequently guiding bone regeneration. To further investigate the internal vascular distribution in the early stages of Gyroid-shaped scaffold implantation, previous studies primarily employed micro-CT three-dimensional imaging after arterial perfusion with contrast agents.^57^ However, the titanium alloy material is prone to artifact interference, making it difficult to accurately assess the vascular distribution within the scaffold. Therefore, this study attempted a regionalized approach for sample analysis. Following the above method, we combined regional sampling with Western blotting and found that CD31 and EMCN expression levels were significantly higher near the upper bone defect site than in the mandibular border region, potentially related to the inducing effect of the bone defect. Notably, though titanium implants introduced artifacts in imaging assessments, cone-beam computed tomography (CBCT) clearly demonstrated successful implantation and stability of all scaffold structures. Concurrently, we endeavored to minimize artifact effects. Quantitative analysis of imaging data yielded new bone volume results consistent with histological staining, further confirming the significant osteogenic potential of the Gyroid-shaped TC4 porous bioscaffold.

Kusumbe et al. discovered that hypoxia-inducible factor 1α (HIF-1α) is a key transcription factor inducing H-type angiogenesis, thereby promoting bone regeneration and repair processes.^58^ Similarly, vascular endothelial growth factor A (VEGFA) serves as a crucial regulator in angiogenesis, modulating endothelial cell migration, proliferation, and lumen formation during bone defect repair.^59^ We found through RT-qPCR that the gene expression levels of HIF-1α and VEGFA in Gyroid-shaped scaffolds were significantly higher than those in Cube-shaped scaffolds, with Gyroid-shaped scaffolds exhibiting higher gene expression levels even in the early stages of implantation. Notably, after 2 weeks of implantation, no significant difference in HIF-1α and VEGFA expression was observed between the two scaffold types, likely due to the cube scaffold reaching a temporary peak expression at this time point. This overall trend aligns with Western Blot results, further confirming the superior angiogenesis potential of the Gyroid-shaped TC4 porous bioscaffold. Moreover, its vascular formation mechanism likely involves hypoxia-induced upregulation of HIF-1α and VEGFA genes. As reported, osteoblasts and endothelium cells upregulated HIF-1α expression under hypoxic conditions, thereby promoting VEGFA transcription and release—a key regulatory pathway for angiogenesis.^60,61^ Thus, scaffold geometry may also influence cellular fate (Figure 8). Yang et al. discovered that the Gyroid structure’s unique hyperbolic topology could alter cell morphology, induce cytoskeletal reorganization and nuclear deformation, activate the FAK/MAPK signaling pathway, guide the osteogenic differentiation and paracrine angiogenesis of bone marrow-derived mesenchymal stem cells, and accelerate both angiogenesis and osteogenesis.^62^ Li et al. also reported that the curved pores of Gyroid structures promoted HUVECs survival and proliferation. Through mechanotransduction, they enhanced the YAP/TAZ pathway, significantly boosting vascular endothelial cell migration and microtubule formation.^31^ In summary, the geometric morphology of Gyroid-shaped scaffolds plays a pivotal role in both angiogenesis and osteogenesis.

**Figure 8:**
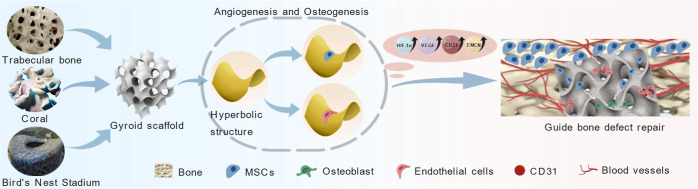
Novel Gyroid-shaped TC4 porous scaffolds guide potential pathways for angiogenesis and osteogenesis.

## 5 Conclusions

In summary, this study successfully fabricated 4 mm single-cell-sized Gyroid-shaped and Cube-shaped TC4 porous bioscaffolds using selective laser melting (SLM) and sand-blasting acid-etching (SLA) techniques. The Gyroid-shaped TC4 porous scaffold, with its unique topological structure, high specific surface area, and excellent biocompatibility, effectively promotes early angiogenesis in bone defect areas, thereby facilitating bone defect repair. This angiogenesis may be associated with the high expression of hypoxia-inducible factor 1α (HIF-1α) and vascular endothelial growth factor A (VEGFA) genes. This provides theoretical reference for the structural design of bone repair materials.

## Supplementary Material



## Notes

For Supporting Information regarding this experiment, the following aspects are primarily covered: Images of the software design (Figure S1), 3D-printed rabbit mandible resin model, porous scaffold structure (Figure S2), acid etching experiments, and most experimental equipment (Figure S3,S4). Abbreviations (Table S1) (PDF)
