# Supplementary material for: An Experimental Study on 3D-Printed Gyroid-Shaped TC4 Porous Scaffolds Guiding Angiogenesis and Osteogenesis in Bone Defect Areas
Source: ACS Biomater Sci Eng. 2026 Jan 9;12(2):1191–203. doi: 10.1021/acsbiomaterials.5c01845 (PMC12892247; doi:10.1021/acsbiomaterials.5c01845)
Supplement: Supplementary file 1 [file ab5c01845_si_001.pdf]

Supporting Information for Publication:

An experimental study on 3D-printed Gyroid-shaped TC4 porous scaffolds guiding angiogenesis and osteogenesis in bone defect areas

Lei Wang [a,b,c], *Yu Wang* [a,b], *Rui Liu* [a,b], *Yanfeng Liang* [c], *Yang Liu* [a,b], *Mingqi Xu* [a,b], *Jia Yu* [a,b], *Yucheng Su* [d], *Zekui Han* [a,b,*], *and Xinyu Wang* [a,b,*]

a Key Laboratory of Oral Biomedical Materials and Clinical Application, School of Stomatology, Jiamusi University, 522 Hongqi Street, Jiamusi, 154002, China

b Experimental Center of Stomatology Engineering, School of Stomatology, Jiamusi University, 522 Hongqi Street, Jiamusi, 154002, China

c Life Science Key Laboratory Center of Basic Medicine, School of Basic Medicine, Jiamusi University, 258 Xuefu Street, Jiamusi, 154007, China

d Beijing Implant Training College (BITC), 109 Xidan North Street, Beijing, 100032, China

S2

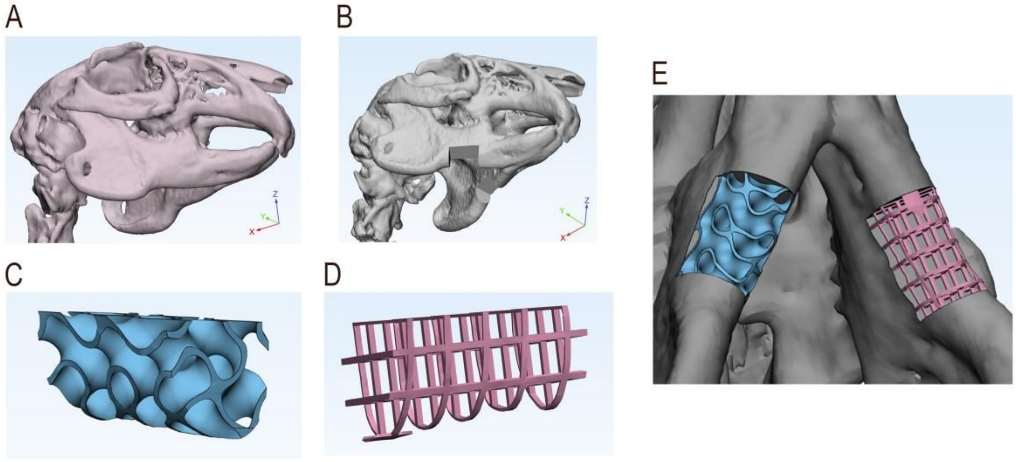

Figure S1: Software Design Images

(A) Rabbit mandible; (B) Bilateral mandibular defects; (C) Gyroid-shaped porous scaffold; (D) Cube-shaped porous scaffold; (E) Merged bone defect and scaffold structure;

S3

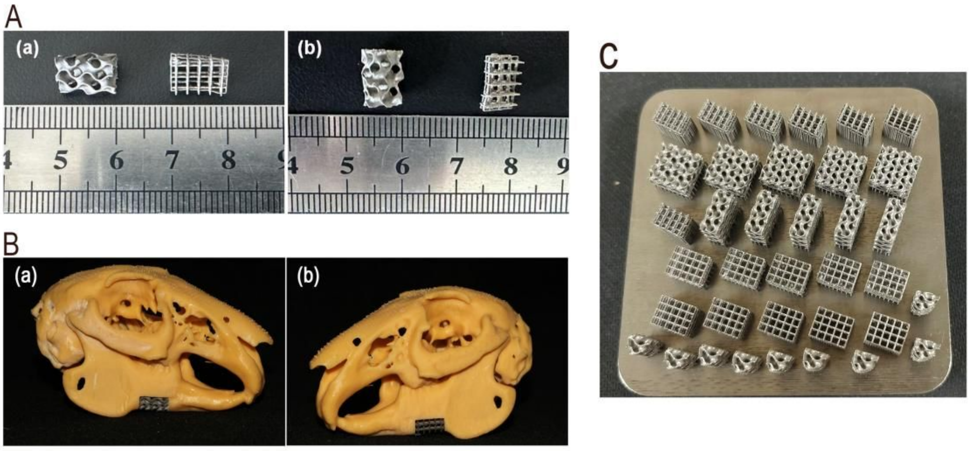

Figure S2: 3D-Printed Mandible Model and Porous Scaffold Structures

(A) Gyroid-shaped and Cube-shaped TC4 porous scaffolds;

(B) Resin model of rabbit mandible and scaffold structures;

(C) 3D-printed scaffold structures (Substrates)

S4

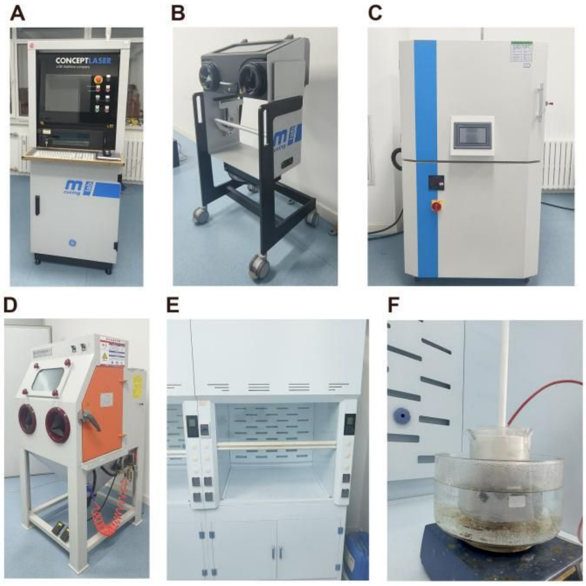

Figure S3: Metal 3D Printing Equipment and Acid Etching Process

(A) Metal 3D printer; (B) Glove box; (C) Annealing furnace; (D) Sandblasting machine; (E) PP ventilation hood; (F) Acid etching image;

S5

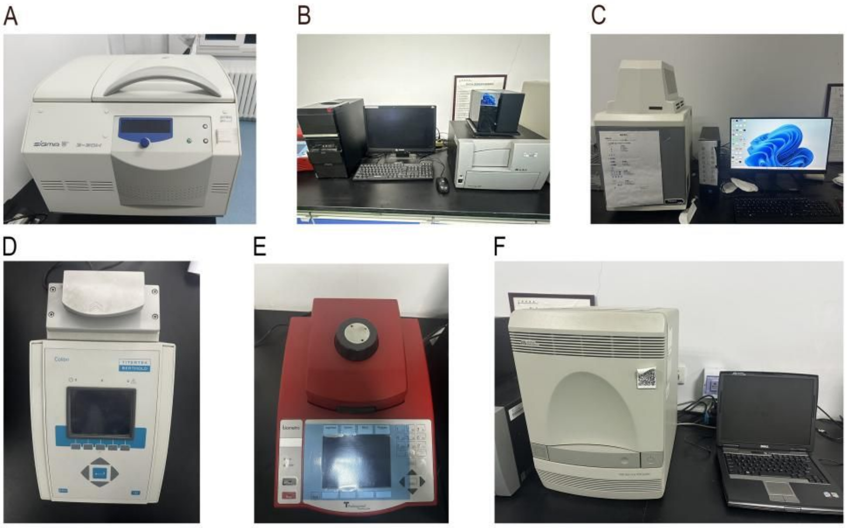

Figure S4: Biological Detection Equipment

(A) Low-temperature high-speed centrifuge; (B) Fully automated microplate reader; (C) Fully automated chemiluminescence imaging system; (D) Ultraviolet spectrophotometer; (E) Reverse transcription instrument; (F) 7300 PCR reaction system;

S6

Table S1: Abbreviations

Full name	Abbreviation
Beijing Implant Training College	BITC
Titanium alloy	TC4
Selective Laser Melting	SLM
Ultrasonic Cleaning	UC
Sandblasting	SA
Sandblasting and Acid Etching	SLA
Alumina	Al_2O_3
Field Emission Scanning Electron Microscopy	SEM
Energy-Dispersive X-ray Spectroscopy	EDS
Phosphate-Buffered Saline	PBS
Hypoxia-Inducible Factor	HIF
Hypoxia-Inducible Factor 1α	HIF-1α
Vascular Endothelial Growth Factor A	VEGFA
Platelet Endothelial Cell Adhesion Molecule-1	CD31
Endothelial Mucin	EMCN
Three-Dimensional Printing	3D Printing
Hematoxylin-Eosin staining	H&E Staining
Regions of Interest	ROI
SDS Polyacrylamide Gel Electrophoresis	SDS-PAGE
Electrochemiluminescence	ECL
Cone-Beam Computed Tomography	CBCT
The New Bone Volume Fraction	NBV/TV
Standard Deviation	SD
